# Dietary intake of total polyphenols and the risk of all-cause and specific-cause mortality in Japanese adults: the Takayama study

**DOI:** 10.1007/s00394-019-02136-9

**Published:** 2019-11-15

**Authors:** Chie Taguchi, Yoshimi Kishimoto, Yoichi Fukushima, Kazuo Kondo, Michiyo Yamakawa, Keiko Wada, Chisato Nagata

**Affiliations:** 1 Endowed Research Department ‘Food for Health’, Ochanomizu University, 2-1-1 Otsuka, Bunkyo-ku, Tokyo, 112-8610 Japan; 2 Nestlé Japan Ltd, Tokyo, Japan; 3 Faculty of Food and Nutritional Sciences, Toyo University, Gunma, Japan; 4 Department of Epidemiology and Preventive Medicine, Gifu University Graduate School of Medicine, Gifu, Japan

**Keywords:** Polyphenol intake, Mortality, Cardiovascular disease, Stroke, Prospective cohort study

## Abstract

**Purpose:**

Several epidemiological studies have demonstrated the health benefits of polyphenols, but the associations between polyphenol intake and mortality including total and major causes of death remain unclear. We investigated the associations between subjects’ total polyphenol intake and their mortality from all causes, cardiovascular disease (CVD), cancer, and other causes of death in a population-based cohort study in Japan.

**Methods:**

A total of 29,079 residents of Takayama City, Japan were analyzed. Their dietary intake was assessed using a validated semi-quantitative food-frequency questionnaire (FFQ) in 1992. Mortality was ascertained over the subsequent 16 years. The dietary polyphenol intake was calculated by matching the subjects’ food consumption data with our original polyphenol content database.

**Results:**

A total of 5339 deaths occurred during the follow-up. After multivariable adjustment, the highest quartile of total polyphenol intake compared with the lowest quartile was significantly associated with a lower risk of all-cause mortality (HR: 0.93, 95% CI: 0.82–0.99, *p* trend = 0.003). The subjects in the highest quartile showed significantly lower CVD mortality compared to those in the lowest quartile, and among the types of CVD mortality, a strong inverse association was observed for stroke mortality. Inverse associations were also observed for mortality from other causes, specifically digestive disease. The total polyphenol intake was not significantly associated with the risk of cancer mortality.

**Conclusions:**

The results of this prospective study indicate that dietary total polyphenol intake in Japanese is inversely associated with all-cause mortality and mortality from cardiovascular and digestive diseases.

## Introduction

Polyphenols are the largest group of phytochemicals, and they are present in most foods and beverages of plant origin. Multiple functions of polyphenols, such as antioxidant, anti-inflammatory, anti-microbial, pro-apoptotic activity, and regulations of glucose and lipid metabolism and vascular function, are thought to contribute to human health [1, 2]. The very first reports of possible associations between polyphenol consumption and subsequent disease risk involved the Zutphen Elderly Study [3, 4] and the Seven Countries Study [5] in the 1990s. However, those studies included a limited number of polyphenols in the dietary assessment, reflecting the lack of extensive polyphenol content data at the time. More recent epidemiological studies used a holistic polyphenol database such as the Phenol-Explorer database [6]. Several studies have estimated dietary intake of total polyphenol and suggested that polyphenol consumption contributes to a reduction in the risks of chronic diseases such as cardiovascular diseases (CVDs) [7, 8] and some cancers [9], in addition to all-cause mortality [10, 11]. However, the relationship between dietary total polyphenol intake and mortality has not been studied in a Japanese population or in Asian countries.

Since Japan has a unique food culture, we established an original database for the polyphenol content of foods consumed in Japan to precisely estimate the daily polyphenol intake by Japanese individuals. Using the database, we have estimated the total polyphenol intake in several Japanese populations [12–17]. The intake patterns of polyphenol vary among countries; Japanese consume polyphenols largely from beverages (approx. 80%), and less from vegetables and fruits (< 10%) according to our studies [13, 15–17], whereas not only beverages but also vegetables and fruits contribute to polyphenol intake in some studies conducted in European countries [18–20]. In addition, the mortality rates and contributing risk factors for the onset of chronic diseases such as CVD and cancer also vary from country to country. It is, therefore, important to evaluate the relationship between dietary polyphenol intake and the risk of death in each population. We conducted the present study to examine the relationships between dietary total polyphenol intake and all-cause and cause-specific mortalities in a Japanese population.

## Methods

### Study population

The study subjects were members of the Takayama Study cohort [21]. The Takayama Study, established in 1992, included 14,427 male and 17,125 female residents of Takayama, Gifu Prefecture, Japan, aged ≥ 35 years. Those subjects completed a baseline self-administered questionnaire, yielding a participation rate of 85.3%. The present study included 29,079 subjects (13,355 men and 15,724 women) with no history of cancer, stroke, or ischemic heart disease (IHD). The details of the study design and subjects have been described [21].

### Dietary and non-dietary assessments

In 1992, the subjects completed a baseline self-administered questionnaire that included questions on their demographic characteristics, smoking status, diet, physical activity, and medical and reproductive histories. Diet, including alcohol intake, was assessed with the use of a 169-item semi-quantitative food-frequency questionnaire (FFQ) that includes 520 food items. The FFQ asked the subjects to describe their usual frequency of consumption and usual serving size of each item during the 12 months prior to filling out the FFQ. The detailed information on the FFQ including its validity and reproducibility has been published [22]. Physical activity was assessed by asking the mean number of hours per week spent performing various types of physical activity during the past 12 months. The mean number of hours per week spent at each intensity of activity was multiplied by its correspondent energy expenditure requirements, expressed as a metabolic equivalent, and summed up to yield a score (metabolic equivalent, h/week). The details of the questionnaire, including its validity, are described elsewhere [23].

For the estimation of dietary total polyphenol intake, we used our original database of the polyphenol content of foods; the main values from this database are described elsewhere [11, 12]. In this database, total polyphenol contents were calculated as catechin equivalents, except for coffee whose content was calculated as chlorogenic acid equivalents. Foods that contain no or only traces of polyphenols were excluded from the estimation. The total polyphenol content in each food was measured using a modified Folin–Ciocalteu method using reverse-phase column chromatography to remove the interference by nonpolyphenol compounds [24]. For foods without the analytical value, the value of similar foods or a different form of the same food was assigned. For processed foods made of polyphenol-containing ingredients (e.g., wheat flour, soy sauce, sweet bean paste), the polyphenol contents were calculated by the values of the ingredients and their proportion of the total food component. The coverage of our database on plant origin food items including in the FFQ was 98%. We calculated the daily polyphenol intake by matching the food consumption data from each subject’s completed FFQ with the polyphenol content in the foods.

### Follow-up and endpoints

Deaths and their causes that occurred in this cohort between the baseline (September 1, 1992) and October 1, 2008 were confirmed from death certificates provided by Japan’s Legal Affairs Bureau. The causes of deaths were coded according to the International Classification of Diseases, Tenth Revision (ICD–10). The endpoints were all-cause mortality and disease-specific mortalities including cancer (ICD-10: C00–D48), CVD (ICD-10: I00–I99) and all other causes (non-cancer, non-CVD). Information concerning subjects who died or moved away from Takayama City during the course of the study was obtained from residential registers or family registers.

During the study period, 1912 individuals (6.1%) moved out of Takayama City. The date of emigration was unknown for 104 subjects (0.8%). They were censored at the latest date on which they were known to reside in the city. This study was conducted according to the guidelines laid down in the Declaration of Helsinki and all procedures involving human subjects were approved by the ethical board of the Gifu University Graduate School of Medicine. Written informed consent was obtained from all subjects.

### Statistical analysis

We calculated person-years from the date of the subject’s response to the baseline questionnaires to the date of subject’s death, the date of the subject’s emigration out of Takayama, or the end of the follow-up (October 1, 2008), whichever occurred first. The total polyphenol intake was energy-adjusted by the residual method proposed by Willett [25]. We divided the subjects into quartiles according to their energy-adjusted dietary intake of total polyphenol. We calculated the hazard ratio (HR) and 95% confidence interval (95% CI) for all-cause mortality and cause-specific mortalities for each category using the Cox proportional hazard model in comparison with the lowest intake category. Linear trend tests for the associations were based on median values for each quartile, and this variable was included in the models as a continuous variable. Covariates included in the multivariable models were age, sex, body mass index (BMI) (by quintile), physical activity score, smoking status (never, former, current with ≤ 30 years of smoking, or current with > 30 years of smoking), level of education (≤ 11, 12–14 years, or ≥ 15 years), marital status (married or not married), history of diabetes and hypertension (yes or no), alcohol consumption (by quartile), and dietary factors including energy and intake of salt. Total polyphenol intake was also analyzed as continuous, after a log_2_ transformation, with the HR indicating the increase in the risk of death with a doubling of total polyphenol intake (mg/day). Interactions between sex, age, BMI (by quintile), smoking status (never, former, or current smokers), and total polyphenol intake in relation to mortality were evaluated using a likelihood ratio test based on models with and without an interaction term. As a sensitivity analysis, we conducted competing risk regression analyses using the method proposed by Fine and Gray [26]. The association of the polyphenol intake with CVD, stroke and digestive disease mortality was assessed with non-CVD, non-stroke, and non-digestive disease death as a competing event, respectively. All statistical analyses were conducted with SAS software, ver. 9.4. Statistical significance was defined as a two-sided *p* value < 0.05.

## Results

The subjects consumed 759 ± 410 mg/day of polyphenols on average (18–4176 mg/day). The baseline characteristics of the subjects according to quartile of total polyphenol intake are summarized in Table 1. The subjects in the highest quartiles of polyphenol intake were more likely to be young, married, higher-educated, smokers, and more physically active, and less likely to have hypertension and diabetes. There were no significant differences in energy intake among the four quartiles. The difference in the average of total polyphenol intake between Q1 and Q4 was 3.5-fold, and this difference closely reflected that of the subjects’ polyphenol intake from beverages. The difference in the average of polyphenol intake from food among the quartiles was small, i.e., within a narrow range of 243–277 mg/day.

**Table 1:** Baseline characteristics of study subjects according to quartiles of total polyphenol intake (*n* = 29,079)

	Quartiles of total polyphenol intake (energy-adjusted) (mg/day)			
	Q1 < 501.9	Q2 501.9–705.7	Q3 705.7–951.6	Q4 > 951.6
*n*	7270	7270	7270	7269
Age, years	58.9 ± 12.4	56.4 ± 12.9	54.7 ± 12.7	48.4 ± 9.9
Female, %	44.5	55.9	61.8	54.2
Married, %	81.5	81.1	81.6	86.5
Years of education, %				
≤ 11	76.1	67.3	59.0	45.7
12–14	19.7	26.6	32.4	41.2
≥ 15	4.1	6.1	8.5	13.1
Smoking, %				
Never	49.1	55.1	56.7	43.0
Former	20.9	17.3	14.4	11.1
Current	29.6	27.8	28.9	45.9
History of hypertension, %	23.3	20.2	17.3	11.5
History of diabetes, %	4.7	4.1	4.1	3.8
Height, cm	157.4 ± 9.2	157.2 ± 9.3	157.1 ± 9.0	159.8 ± 8.7
BMI, kg/m^2^	22.2 ± 2.9	22.1 ± 2.9	22.2 ± 2.8	22.3 ± 2.8
Exercise, METs-h/week	21.3 ± 35.5	21.6 ± 34.7	23.3 ± 36.3	24.2 ± 36.6
Alcohol intake, mg/day	27.3 ± 39.1	21.8 ± 33.4	20.0 ± 31.8	24.9 ± 35.3
Dietary intake				
Total energy, kcal/day	2435 ± 931	2306 ± 799	2243 ± 820	2444 ± 877
Polyunsaturated fat, g/day	14.8 ± 7.7	14.4 ± 6.7	14.4 ± 6.6	15.5 ± 7.0
Dietary fiber, g/day	16.0 ± 8.8	16.1 ± 8.6	16.5 ± 8.6	17.2 ± 9.3
Salt, g/day	13.6 ± 6.6	13.3 ± 6.0	13.4 ± 5.9	14.1 ± 6.2
Polyphenol intake (energy-adjusted), mg/day				
Total (beverages + foods)	357 ± 104	612 ± 58	816 ± 72	1250 ± 266
Beverages	114 ± 106	352 ± 87	539 ± 106	976 ± 278
Coffee	37 ± 57	113 ± 124	188 ± 163	614 ± 322
Green tea	31 ± 74	166 ± 138	252 ± 145	219 ± 160
Other beverages	47 ± 65	73 ± 88	99 ± 109	143 ± 154
Foods	243 ± 74	261 ± 73	277 ± 78	274 ± 92
Seasoning	71 ± 24	72 ± 22	74 ± 23	72 ± 24
Vegetables	51 ± 30	55 ± 31	59 ± 33	57 ± 38
Fruits	33 ± 32	40 ± 34	46 ± 39	48 ± 50
Pulses	28 ± 19	29 ± 20	30 ± 20	27 ± 21
Other foods	61 ± 25	65 ± 25	68 ± 25	69 ± 29

Values are mean ± SDs or %

*BMI* body mass index, *MET* metabolic equivalent

We identified 5339 deaths during the mean follow-up period of 14.1 years. Table 2 shows the HRs and 95% CIs for mortality by quartiles of total polyphenol intake. After the multivariable adjustment, the subjects in the highest quartile had a lower all-cause mortality rate compared to those in the lowest quartile (HR 0.93, 95% CI: 0.82–0.99, *p* trend = 0.003). The HR for log_2_ was 0.94 (95% CI: 0.91–0.98) when total polyphenol intake was included as a continuous variable, suggesting that a doubling of the total polyphenol intake was associated with a 6% decrease in all-cause mortality rate. The total polyphenol intake was not significantly associated with the risk of cancer mortality. The subjects in the highest quartile presented significantly lower CVD mortality compared to those in the lowest quartile (HR 0.83, 95% CI: 0.70–0.99, *p* trend = 0.009), and the HR for log_2_ was 0.93 (95% CI: 0.87–0.99). After stratification by the type of CVD, the highest quartile of total polyphenol intake, compared with the lowest quartiles, was significantly associated with a decreased risk of mortality from total stroke (HR 0.69, 95% CI: 0.51–0.94, *p* trend = 0.02), but not associated with the risk of IHD. For other causes of mortality, the HRs for the highest versus lowest quartile of total polyphenol intake were 0.84 (95% CI: 0.72–0.98, *p* trend = 0.004). Regarding the associations between total polyphenol intake and mortality from other specific causes, total polyphenol intake was strongly associated with a lower risk of mortality from digestive disease (highest versus lowest quartile HR 0.36, 95% CI: 0.18–0.70, *p* trend = 0.001, and HR for log_2_ 0.76, 95% CI: 0.62–0.92). Total polyphenol intake was also tended to be inversely associated with mortality from respiratory disease (HR 0.81, 95% CI: 0.61–1.07, *p* trend = 0.08) and infectious disease (HR 0.59, 95% CI: 0.31–1.10, *p* trend = 0.09), although the point estimates were not statistically significant. There was no substantial effect modification by sex, age, BMI, and smoking status for the mortality from all-cause, CVD, stroke and digestive disease.

**Table 2:** Hazard ratios (HRs) with 95% CIs for mortality by quartiles of total polyphenol intake in study subjects

	Quartiles of total polyphenol intake (energy-adjusted) (mg/day)					Continuous (log_2_)
	Q1 < 501.9	Q2 501.9–705.7	Q3 705.7–951.6	Q4 > 951.6	*p* trend	
*n*	7270	7270	7270	7269		
All causes						
No. of cases	1940	1514	1206	679		
Age and sex-adjusted HR	1.0	0.94 (0.88–1.00)	0.88 (0.82–0.95)	0.97 (0.89–1.06)	0.08	
Multivariable HR^a^	1.0	0.93 (0.86–0.99)	0.87 (0.81–0.94)	0.93 (0.82–0.99)	0.003	0.94 (0.91–0.98)
Cancer						
No. of cases	541	425	373	281		
Age-adjusted HR	1.0	0.95 (0.83–1.08)	0.96 (0.84–1.10)	1.12 (0.96–1.30)	0.23	
Multivariable HR^a^	1.0	0.93 (0.82–1.06)	0.92 (0.80–1.05)	1.01 (0.87–1.18)	0.92	1.00 (0.93–1.07)
Cardiovascular disease						
No. of cases	623	496	390	169		
Age and sex-adjusted HR	1.0	0.93 (0.82–1.04)	0.86 (0.76–0.98)	0.88 (0.74–1.05)	0.04	
Multivariable HR^a^	1.0	0.92 (0.81–1.03)	0.85 (0.75–0.97)	0.83 (0.70–0.99)	0.009	0.93 (0.87–0.99)
Stroke						
No. of cases	252	207	163	55		
Age and sex-adjusted HR	1.0	0.97 (0.80–1.16)	0.91 (0.74–1.11)	0.71 (0.53–0.96)	0.03	
Multivariable HR^a^	1.0	0.97 (0.81–1.17)	0.91 (0.74–1.11)	0.69 (0.51–0.94)	0.02	0.94 (0.85–1.04)
Ischemic heart disease						
No. of cases	101	88	71	48		
Age and sex-adjusted HR	1.0	1.02 (0.77–1.36)	0.96 (0.71–1.31)	1.29 (0.90–1.83)	0.28	
Multivariable HR^a^	1.0	0.98 (0.73–1.31)	0.92 (0.67–1.25)	1.13 (0.78–1.63)	0.71	0.96 (0.83–1.12)
All other causes						
No. of cases	776	592	442	228		
Age and sex-adjusted HR	1.0	0.93 (0.84–1.04)	0.83 (0.74–0.94)	0.88 (0.76–1.02)	0.01	
Multivariable HR^a^	1.0	0.92 (0.82–1.02)	0.84 (0.75–0.95)	0.84 (0.72–0.98)	0.004	0.91 (0.86–0.96)
Respiratory disease						
No. of cases	289	200	154	66		
Age and sex-adjusted HR	1.0	0.89 (0.74–1.07)	0.86 (0.71–1.05)	0.87 (0.66–1.14)	0.15	
Multivariable HR^a^	1.0	0.85 (0.70–1.02)	0.86 (0.70–1.05)	0.81 (0.61–1.07)	0.08	0.89 (0.81–0.98)
Injury						
No. of cases	125	94	87	64		
Age and sex-adjusted HR	1.0	0.91 (0.69–1.19)	0.96 (0.73–1.26)	0.93 (0.68–1.27)	0.70	
Multivariable HR^a^	1.0	0.93 (0.71–1.22)	1.01 (0.76–1.34)	0.96 (0.69–1.32)	0.88	1.03 (0.89–1.19)
Digestive disease						
No. of cases	66	54	32	11		
Age and sex-adjusted HR	1.0	0.95 (0.66–1.37)	0.65 (0.42–0.99)	0.37 (0.20–0.72)	0.001	
Multivariable HR^a^	1.0	0.97 (0.67–1.40)	0.68 (0.44–1.05)	0.36 (0.18–0.70)	0.001	0.76 (0.62–0.92)
Infectious disease						
No. of cases	57	42	34	13		
Age and sex-adjusted HR	1.0	0.87 (0.58–1.30)	0.82 (0.53–1.26)	0.60 (0.33–1.12)	0.10	
Multivariable HR^a^	1.0	0.85 (0.57–1.28)	0.83 (0.53–1.28)	0.59 (0.31–1.10)	0.09	0.85 (0.68–1.06)

^a^Adjusted for age, sex, total energy, BMI, physical activity, smoking status, education, marital status, histories of diabetes and hypertension, alcohol consumption, and intake of salt

A competing risk analysis demonstrated a similar pattern of results; the HRs of CVD, stroke, and digestive disease mortality for the highest versus lowest quartiles were 0.82 (95% CI: 0.68–0.98, *p* trend = 0.05), 0.68 (95% CI: 0.50–0.93, *p* trend = 0.07) and 0.35 (95% CI: 0.17–0.71, *p* trend = 0.01), respectively. An exclusion of deaths that occurred during the first 3 years of the follow-up period as a sensitivity analysis did not substantially alter the results; the HRs for all-cause and stroke mortality for the highest compared with the lowest quartile of total polyphenol intake were 0.91 (95% CI: 0.82–1.01, *p* trend = 0.008) and 0.70 (95% CI: 0.50–0.97, *p* trend = 0.04), respectively. In further sensitivity analysis, the exclusion of participants with energy intake < 600 kcal and > 4000 kcal did not substantially alter the associations between overall mortality and total polyphenol intake; the HRs for all-cause and stroke mortality for the highest compared with the lowest quartile of polyphenol intake were 0.90 (95% CI: 0.82–0.99, *p* trend = 0.006) and 0.68 (95% CI: 0.50–0.92, *p* trend = 0.02), respectively.

## Discussion

We examined the relationships between dietary total polyphenol intake and all-cause and cause-specific mortalities in a population-based cohort in Japan. Our present findings revealed that the total polyphenol intake, estimated on the basis of the Folin–Ciocalteu method, was associated with a lower risk of all-cause mortality and mortality from CVD (including stroke) and digestive disease.

A prospective observational study within the PREDIMED trial demonstrated that a greater intake of polyphenols was associated with a decreased risk of CVD events and CVD mortality [7]. In addition, a reanalysis of the PREDIMED trial of the subjects at high risk for CVD (*n* = 7447, 4.8 years of follow-up) found a 37% reduction in all-cause mortality in the highest quintiles of total polyphenol intake that calculated as the sum of individual polyphenols using the Phenol-Explorer database [11]. A prospective Italian cohort study showed that the overall mortality was reduced by 30% in the subjects in the highest tertile of total polyphenol urine excretion (a biomarker of total polyphenol intake) among older adults (≥ 65 years, *n* = 807, 12 years of follow-up) [10]. In the Nutrinet-Santé French cohort study, the researchers found a significant lower cardiovascular disease risk in the highest tertiles of total polyphenol intake when they estimated it on the basis of the Folin–Ciocalteu method [8]. Although we calculated the total polyphenol intakes using our original database based on the Folin–Ciocalteu method, our present finding that a high total polyphenol intake was associated with a lower risk of all-cause and CVD mortality is consistent with these results from European countries. Similarly, in a U.S. cohort study, flavonoid consumption that was analyzed by using the U.S. Department of Agriculture (USDA) flavonoid database (including only flavonoid polyphenolic compounds) was associated with a lower risk of death from CVD [27]. Among the types of CVD mortality, we observed a 31% reduction of stroke mortality comparing the highest versus lowest quartiles of total polyphenol intake. There was a null association between the total polyphenol intake and IHD mortality.

Although a meta-analysis of prospective cohort studies identified that flavonols associated with a lower stroke risk [28], very few studies have reported an association between total polyphenol intake and the risk of CVD mortality divided into coronary heart diseases (CHDs) and cerebrovascular diseases (including stroke). To our knowledge, the present study is the first to demonstrate the inverse association of total polyphenol intake with stroke mortality risk. As for cancer, we observed null relationships between total polyphenol consumption and cancer mortality. The EPIC-Spain study also found null relationships between dietary flavonoid or lignan intake and mortality from cancer [29]. In contrast, recent cohort study in Italy showed a lower risk for cancer as well as all-cause and cardiocerebrovascular mortality by polyphenol-rich diet [9]. The association between total polyphenol intake and the risk of cancer mortality remains inconclusive.

Our present subjects’ polyphenol consumption was largely attributed to coffee and green tea, which is consistent with our previous studies in several Japanese populations [12–17]. The main polyphenolic compounds of coffee are hydroxycinnamic acids including chlorogenic acids, whereas green tea contains some flavonoids, mainly catechins. In a French cohort study including analyses of separate polyphenol subgroups, higher intakes of polyphenols, especially of anthocyanins, catechins, and flavonols, were found to be strongly associated with a decreased CVD risk [8]. The Spanish cohort from the PREDIMED study showed that a greater intake of polyphenols, especially from lignans, flavanols, and hydroxybenzoic acids, was associated with decreased CVD risk [7]. Our present investigation did not include evaluations of polyphenol subgroups, and we, therefore, cannot conclude which types of polyphenols are associated with the decreased risk of all-cause mortality and CVD mortality. Although the intake patterns and food sources of polyphenol may vary among countries, it is noteworthy that we observed a lower risk of all-cause mortality and CVD mortality in a Japanese population with high polyphenol intake.

The results of many studies support the beneficial effects of polyphenols on the major risk factors for CVD such as hypertension [30, 31] and diabetes [32, 33]. The most persuasive concept regarding the mechanism of lowering blood pressure is an increase in nitric oxide production plus an improvement of endothelial function [34]. The total polyphenol urine excretion (a biomarker of total polyphenol intake) was shown to be related to plasma nitric oxide, which is associated with a reduction in blood pressure levels [35]. Toward the goal of decreasing the risk of type 2 diabetes, several types of polyphenols are known to attenuate the rate of glucose absorption and to improve insulin resistance [33, 36]. Antioxidative properties are another potential mediator of the beneficial effects of polyphenols against CVD. Randomized trials have shown that the consumption of several polyphenols or polyphenol-rich foods lowered the amount of oxidized low-density lipoprotein (LDL) [37, 38]. Taking the findings of the above-cited studies and those of our present investigation, it is apparent that greater polyphenol intake may contribute to a reduction in CVD risk though multifactorial etiological pathways. Protective effects may be related to the various polyphenols, but the underlying combinational mechanism is still unclear. In addition, we found a significant inverse association between total polyphenol intake and mortality from digestive disease. It was reported that coffee and green tea could exert such beneficial effects on liver and bowel diseases [39–43]. Further studies evaluating associations between polyphenol intake and digestive disease are awaited.

Our study has some limitations. The FFQ was designed to measure an individual’s relative intake of nutrients, rather than absolute values. The subjects’ consumption of the various foods was assessed only at baseline, and it was thus not possible to investigate changes in these factors over time. We used our original database on the polyphenol contents of foods consumed in Japan based on a modified Folin–Ciocalteu method for calculating the total polyphenol intake. In this approach, we could not analyze each separate polyphenol subgroup, though it is important to clarify the relationship between the intakes of individual subclasses of polyphenols and all-cause and specific-cause mortality in Japanese adults. The Folin–Ciocalteu method is recognized to be less specific and used for measuring total antioxidant capacity, we thus have included a cleanup step using reverse-phase column chromatography to remove the interference by non-polyphenol compounds in the assay. We consider that our modified Folin–Ciocalteu method is suitable for roughly estimating the overall amounts of polyphenols, and the risk of underestimation/overestimation is low [15]. Our database almost completely covers the contributing foods for dietary polyphenol intake in Japanese individuals. The present study also has strengths: a population-based prospective cohort design, a good participation rate, and a long follow-up period.

In conclusion, the results of the present population-based prospective study provide evidence of a protective effect of dietary polyphenols on all-cause mortality and CVD and digestive disease mortality. Future research in different populations is warranted to confirm this protective effect of total polyphenol intake on the risk of mortality and/or chronic diseases.
