# Correction to: Dietary intake of total polyphenols and the risk of all-cause and specific-cause mortality in Japanese adults: the Takayama study

**DOI:** 10.1007/s00394-019-02155-6

**Published:** 2019-12-11

**Authors:** Chie Taguchi, Yoshimi Kishimoto, Yoichi Fukushima, Kazuo Kondo, Michiyo Yamakawa, Keiko Wada, Chisato Nagata

**Affiliations:** 1 Endowed Research Department ‘Food for Health’, Ochanomizu University, 2-1-1 Otsuka, Bunkyo-ku, Tokyo, 112-8610 Japan; 2 Nestlé Japan Ltd, Tokyo, Japan; 3 Faculty of Food and Nutritional Sciences, Toyo University, Gunma, Japan; 4 Department of Epidemiology and Preventive Medicine, Gifu University Graduate School of Medicine, Gifu, Japan

## Correction to: European Journal of Nutrition 10.1007/s00394-019-02136-9

The original version of this article unfortunately contained a mistake. First sentence of the second paragraph of the section “Dietary and non‑dietary assessments” should read as:

For the estimation of dietary total polyphenol intake, we used our original database of the polyphenol content of foods; the main values from this database are described elsewhere [12, 13].

